# COMPAS: AN APP TO PROMOTE PERSON-CENTERED COMMUNICATION BETWEEN PEOPLE LIVING WITH DEMENTIA AND THEIR CAREGIVERS

**DOI:** 10.1093/geroni/igad104.2809

**Published:** 2023-12-21

**Authors:** Barbara Delacourt, Michè le Masson-Trottier, Jade Dubuc, Catherine Dubé, Ana Inés Ansaldo

**Affiliations:** University of Montreal, Montréal, Quebec, Canada; University of Montréal, Montréal, Quebec, Canada; University of Montréal, Montréal, Quebec, Canada; IUGM research center, Montréal, Quebec, Canada; University of Montréal, Montréal, Quebec, Canada

## Abstract

**Introduction:**

People living with dementia (PWD) experience communication deficits as soon as the early stages of the diseases, deficits which significantly increase over time. In the last stages, constant communication breakdowns lead to reduced exchanges with caregivers, resulting in the isolation of both communication partners. These difficulties have negative impacts on the quality of life of PWD and their caregivers, who themselves face increasing burden. While communication difficulties in PWD are a core issue in care, few interventions to address this issue have been developed. Aims: The present study focused on COMPAs, an app designed to sustain communication between PWD and their caregivers. COMPAS has been shown to trigger emotional communication during the short co-viewing of personalized audiovisual material. Method: This was a pre-post intervention study with COMPAs in 2 long-term care centers. Seventeen caregivers used COMPAs in the context of daily routines over eight weeks with seventeen PWD. Data collection included specific questionnaires and semi-structured interviews to measure effects on communication and caregiver burden. Data analyses combined quantitative and qualitative approaches.

**Results:**

In caregivers, there was a significant improvement in personal achievement at work. Semi-structured interviews showed an improvement in communication in the dyad and a more empathetic approach to caregiving.

**Discussion:**

These results indicate that the communication triggered by COMPAs breaks down communication barriers, by creating positive exchanges through personalized emotionally driven exchanges, while stimulating empathy and personalized interventions. Caregivers see COMPAs as an ecological tool to address communication barriers, while facilitating an empathetic caregiving relationship.

